# Supplementary material for: New Insight into the Potential Protective Function of Sulforaphene against ROS−Mediated Oxidative Stress Damage In Vitro and In Vivo
Source: Int J Mol Sci. 2023 Aug 23;24(17):13129. doi: 10.3390/ijms241713129 (PMC10487408; doi:10.3390/ijms241713129)
Supplement: Supplementary file 1 [file ijms-24-13129-s001.zip › ijms-2546570-supplementary.pdf]

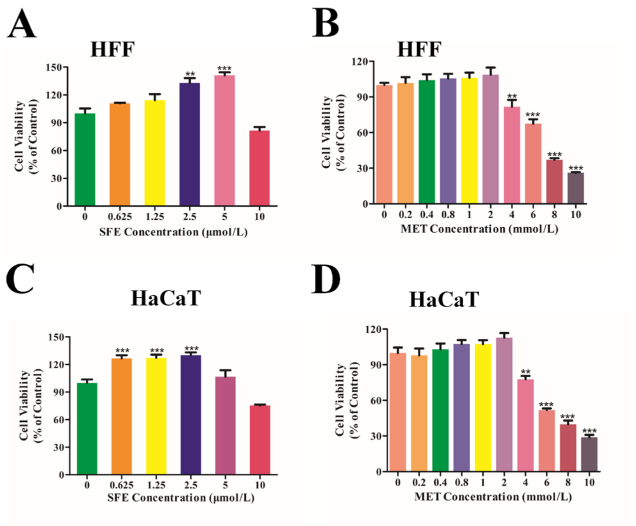

Figure. S1 Effect of SFE on cell viability in HFF and HaCaT cells. (A) HFF cells were pretreated with various concentrations of SFE for 24 h. (B) HFF cells were pretreated with various concentrations of MET for 24 h. (C) HaCaT cells were pretreated with various concentrations of SFE for 24 h. (D) HaCaT cells were pretreated with various concentrations of MET for 24 h. Data are reported as mean ± S.D. (n = 5) for each group. **P < 0.01, ***P < 0.001.
